# Multi-arch dam safety evaluation based on statistical analysis and numerical simulation

**DOI:** 10.1038/s41598-022-13073-9

**Published:** 2022-05-26

**Authors:** Qi He, Chongshi Gu, Silvio Valente, Erfeng Zhao, Xing Liu, Dongyang Yuan

**Affiliations:** 1 State Key Laboratory of Hydrology-Water Resources and Hydraulic Engineering, Hohai University, Nanjing, 210098 China; 2 College of Water Conservancy and Hydropower Engineering, Hohai University, Nanjing, 210098 China; 3 National Engineering Research Center of Water Resources Efficient Utilization and Engineering Safety, Hohai University, Nanjing, 210098 China; 4 Department of Structural, Geotechnical and Building Engineering, Politecnico di Torino, 10129 Turin, Italy; 5 China Three Gorges Construction Engineering Corporation, Chengdu, 610094 China

**Keywords:** Civil engineering, Applied mathematics

## Abstract

The Foziling multi-arch dam, one of the few multi-arch dams in the world, was built on the bedrock with complicated geological conditions. It has undergone several reinforcements since it was put into service in the 1950s. In this study, the dam safety is evaluated by analyzing the measured displacements and simulating stresses in the concrete. Firstly, the multiple linear stepwise regression (MLSR) is used to train and test the relationships between the loads and displacement based on the hydrostatic-temperature-time (HTT) model. Subsequently, the contributions of water level, temperature, and time to displacements are determined, and the influence characteristics of water level and temperature on displacements are interpreted. Finally, the dam stress state is evaluated by establishing a dam finite element model and simulating the stress distribution in various operating conditions. The results indicate that (1) the dam is currently in an elastic state after the last reinforcement; (2) temperature contributes the most to the displacement, and the drastic fluctuation of temperature is the disadvantage factor for multi-arch dam safety; (3) the stresses generally can meet the requirements of code; and (4) the ideas and methods of the study can provide references for the safety evaluation of other concrete dams.

## Introduction

Dams play an important role in water supply, flood control, irrigation, navigation, sedimentation control, and hydropower generation. Dam safety is closely related to property safety, economic development, and the ecological environment in the area around the dam. Dam safety monitoring by instruments is the most important project of dam operation management^1,2^. The data on water level, temperature, stress–strain, and seepage is important monitoring variables in the safety evaluation of concrete dams. The actual response of the structure, such as displacement, is often compared with the model prediction so that the abnormal behavior of the dam can be detected in time and corrective measures can be taken^3–6^.

Displacement observation, which is intuitive and reliable, and can explain the dam behavior itself, has been widely used in practice^1,7,8^. Studies have shown that the displacement is closely related to such factors as water level, temperature, and time. Therefore, establishing an accurate relationship between these factors and displacement can explain and predict dam behavior effectively. At present, there are many models used to describe and predict dam behavior, mainly including statistical models, deterministic models, and mixed hybrid models^9,10^. Statistical models, dating back to the 1950s, are exclusively based on the past monitoring data for explaining the relationship between loads and dam behavior. The statistical models based on multiple linear regression (MLR) and stepwise regression (SR) are widely used for predicting dam behavior due to their simple formulation and fast calculation speed^11,12^. Deterministic models typically explain the behavior of dam on the concept of mechanics based on the finite element method (FEM). However, due to the uncertainty of dam body and foundation materials, it is very difficult to accurately predict dam behavior by FEM. With the development of artificial intelligence technology, machine learning algorithms offer new ways to establish dam behavior prediction models. For instance, it is provided that the models based on the artificial neural network are more accurate in the prediction of the dam displacement than the MLR model^13–15^. The predictive models based on the radial basis function (RBF) network are proved to have the ability to approximate any nonlinear function^16^. Support vector machine shows high accuracy in predicting dam displacement^17,18^. A hybrid model based on a swarm optimized neural fuzzy inference system performed better than multilayer perceptron neural networks and Gaussian processes in forecasting the horizontal displacement of a rockfill dam^19^. And other machine learning algorithms, such as boosted regression trees^20^, extreme learning machines^21^, Random forests^22^, twin support vector regression (TSVR)^23^, and so on, are also used to predict dam behavior. Deep learning is an extension of machine learning. The algorithms represented by Long Short-Term Memory (LSTM) and Gated Recurrent Unit (GRU) are also used to predict dam behavior with the advantages in implicit information mining^24–27^. These cases provide that the nonlinear relationship between input variables and dam response can be better characterized by machine learning algorithms so that the established dam behavior prediction models are more accurate and robust. However, there is a shortcoming that over-fitting may occur. The calculations of these models are in the "black box", and no specific mathematical analytic expressions can be obtained to interpret the physical meaning of dam behavior. It is proved that the hydrostatic-temperature-time (HTT) model is an effective way to reveal how the individual load (such as water level, temperature, and time) influences the dam behavior^28,29^. With the multiple linear stepwise regression (MLSR) method, the parameters of the HTT model can be solved and avoid multicollinearity. Stress observation is another important physical quantity for studying dam operation and evaluating dam safety. But many dam health monitoring facilities are inadequate, and stressometers are damaged or not installed. In dam safety evaluation, the numerical simulation based on finite element models is often used to obtain the actual dam operation behavior^30–34^.

Therefore, the objective of this study is to put forward a method for evaluating the safety of Foziling Muti-arch Dam based on displacements observation and simulated stresses, while the methods can also be applied to other concrete dams. First of all, an HTT displacement model is established based on monitoring data over the past 13 years. And then the contribution and active form of water level, temperature and time to dam displacement are investigated by the MLSR method. Finally, the dam stress states in various operating conditions are evaluated by numerical simulation.

## The Foziling multi-arch dam

### Engineering overview

**Figure 1:**
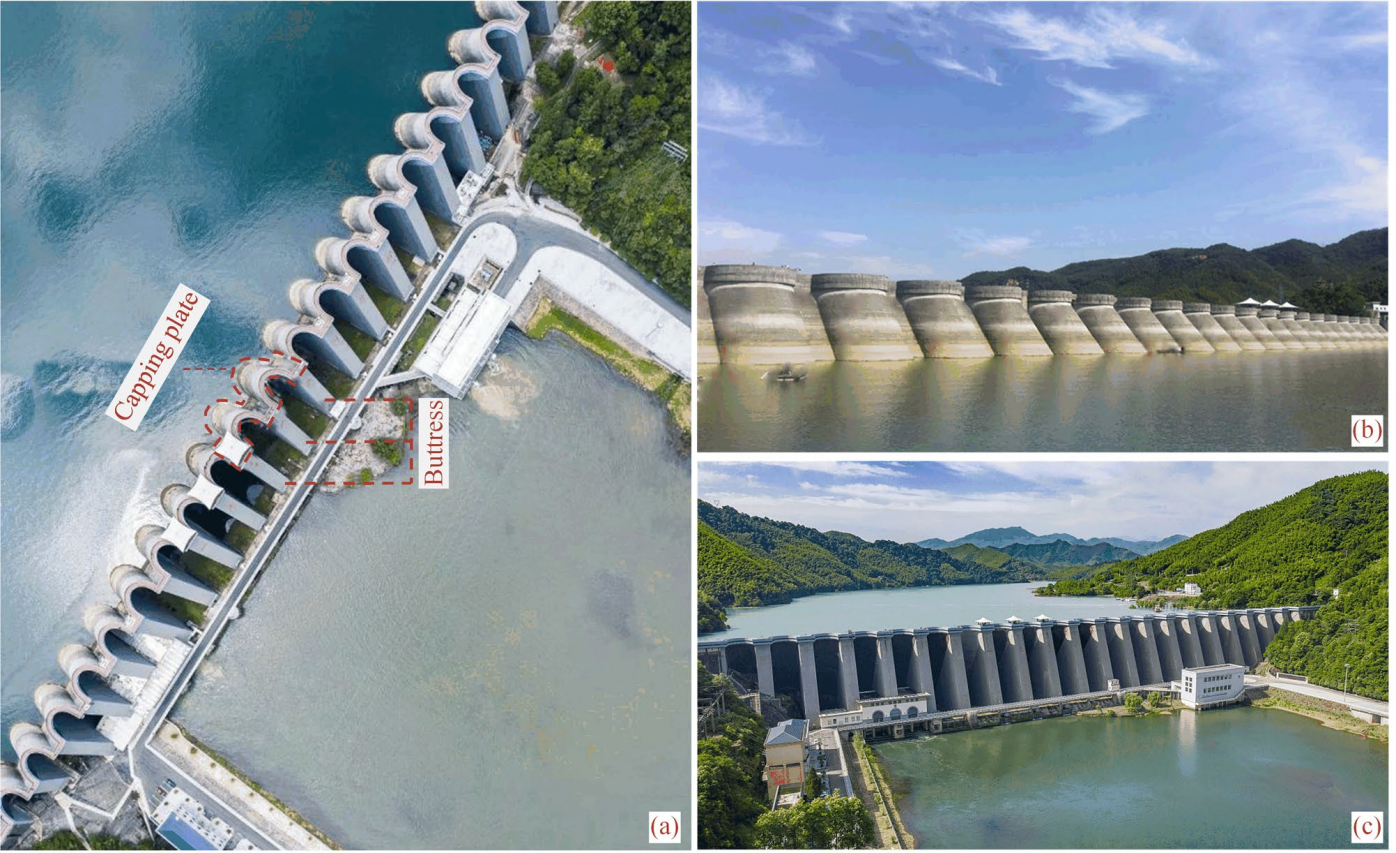
Foziling muli-arch dam: (**a**) top view; (**b**) upstresm view; (**c**) downstream view.

**Figure 2:**
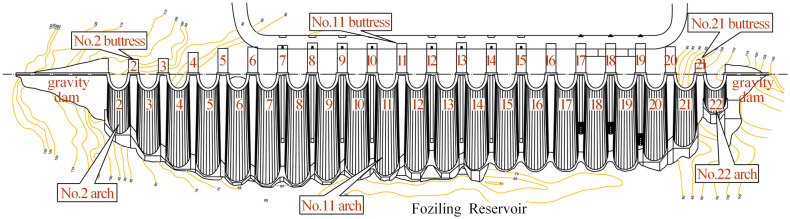
The layout of Foziling multi-arch dam.

The Foziling multi-arch dam, the first reinforced concrete multi-arch dam after the founding of the People's Republic of China, built-in 1952, is located on the Bi River in Anhui Province. It is an annual regulating reservoir with comprehensive utilization benefits of flood control, irrigation, power generation, and water supply. The total storage capacity is 1.94 × 10^8^ m^3^, and the reservoir’s normal storage level, design flood level, and check flood level are 125.56 m, 125.97 m, and 129.80 m, respectively. Figure 1 shows the top, upstream, and downstream views of the dam. The crest of the dam is 510 m long and 1.8 m wide. As is shown in Fig. 2, the Foziling multi-arch dam is composed of 20 buttresses, 21 arches, and the gravity dam sections. The crest elevation is 131.06 m and the maximum dam height is 75.9 m, including the wave wall height of 1.5 m.

**Figure 3:**
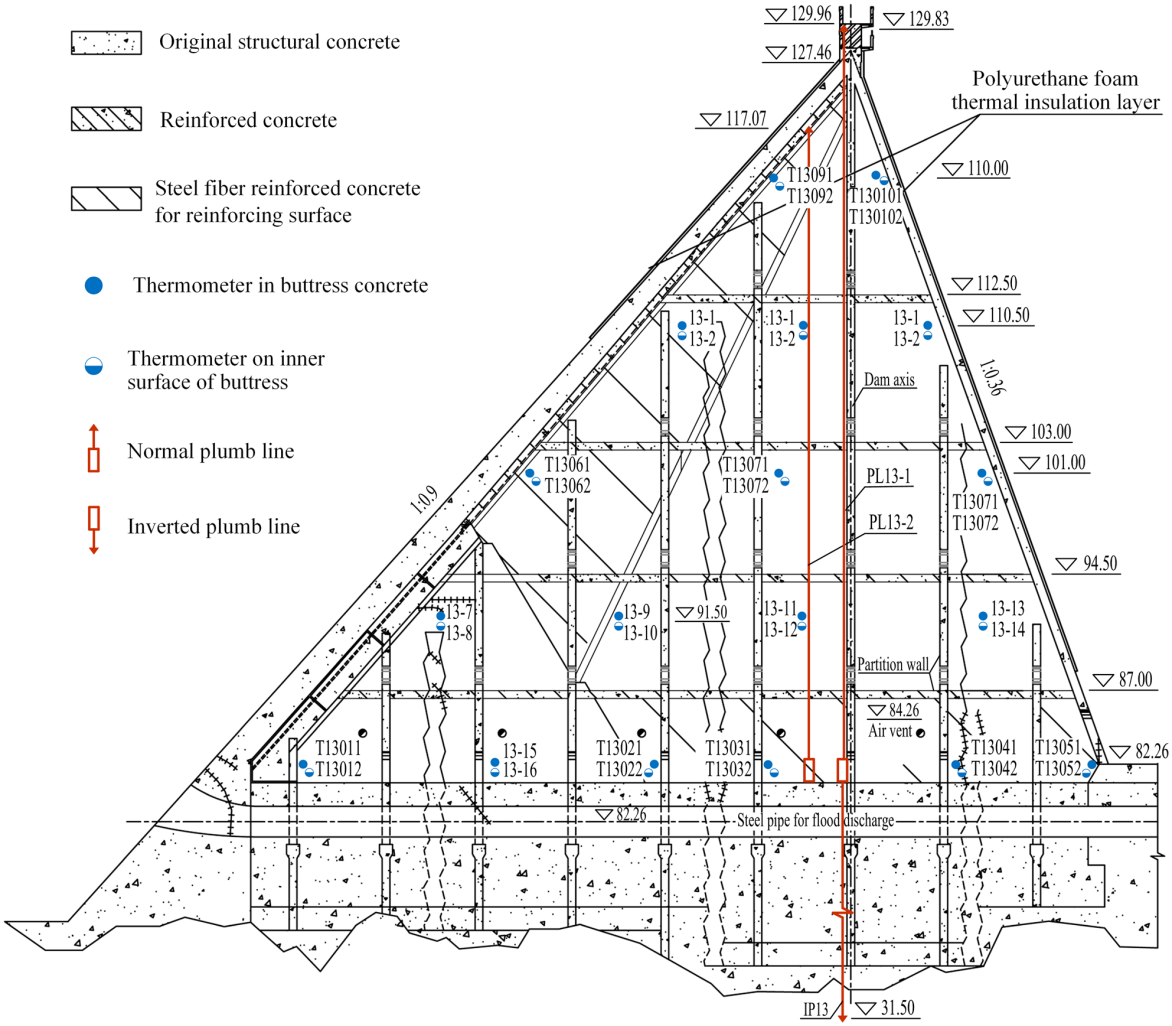
The vertical cross of No.13 buttress (unit: m).

The arches and buttresses of the multi-arch dam are thin-walled and lightweight structures. The buttress is formed by connecting two triangular vertical walls and partition walls, which are shown in Fig. 3. The buttress wall is thin at the top but thick at the bottom, with a thickness of 0.6 m at the crest and 1.97 m at the elevation of 70.0 m away from the crest. The arch section is semicircular with an inner radius of 6.75 m, while the outer radius varies with the thickness of the arch. From the dam crest down, the arch section gradually thickens, with a thickness of 0.6 m at the dam crest and 2.0 m at the elevation of 70.0 m away from the top. Due to the small storage capacity, the large variation range of water level, complex terrain and geological conditions of the dam foundation, and insufficient temperature control during the construction, the dam has undergone three times large-scale maintenance and reinforcement since it was put into service^35^.

The first reinforcement was carried out from June 1965 to October 1968. In order to solve the problems of cavitation and vibration of steel pipes in the buttresses and cracks leakage, the reservoir was emptied, the reinforced arches were poured at the bottom of No. 13–16 arches, the steel pipes were reinforced in the buttresses, and shrinkage joints of each buttress were grouted. In addition, consolidation grouting and curtain reinforcement were carried out on the dam foundation, and dam monitoring facilities were added. Therefore, the flood control standard of the reservoir was improved.

The second reinforcement was carried out from June 1982 to June 1986. Due to the flood overtopping accident in July 1969, in which the highest reservoir water level exceeded the top of the wave wall by 1.08 m, the dam was increased by 1.5 m and one additional spillway was built. Meanwhile, the dam foundation and the right bank dam foundation curtain were reinforced to improve the dam safety.

The third reinforcement was carried out from October 2002 to April 2005. After the flood season in 1993, the reservoir operated at the higher water level for a long time. A sudden cold current occurred in late November, causing the displacement of each buttress to exceed the historical extreme value. Although the dam has been reinforced twice, serious cracks still existed. According to the results of the 2002 safety appraisal, the reservoir safety was rated as Class III, which belongs to the dangerous reservoir and needed reinforcement again. The main reinforcement content can be divided into four parts: (1) Foundation treatment projects, including slag clearance and excavation, micro-expansion concrete backfill in the buttresses, high-pressure consolidation grouting for dam foundation, curtain grouting for upstream dam foundation, were conducted and foundation drainage holes and seepage observation holes were added on both banks. (2) Reinforcement projects, including repair of dam body cracks, thickness reinforcement for the high-stress areas upstream of dam buttresses by cast-in-situ steel fiber reinforced concrete (SFRC), new horizontal reinforced concrete partitions within the buttresses, and spraying SFRC of the upstream and downstream surfaces of No.2 and 22 arches. (3) Polyurethane thermal insulation layer was sprayed in the upstream water level fluctuation zone (upstream elevation above 110 m to the wall) and downstream dam surface (cf. Fig. 3). (4) Dam safety automation monitoring system and other management facilities were added.

### Instrumentations for dam monitoring

Water level fluctuations, dam displacements, and temperature variations including dam concrete, water in front of the dam, and ambient air, were continuously monitored during the operation of the reservoir.

Two thermometers (TQ13_1, TQ13_2) monitoring the air temperature nearby are respectively installed at the front and tail of the cavity formed by the No.13 arch and the buttresses connected to it. Another thermometer (T) is settled in the louver box on the crest of the wave wall of the gravity dam on the right bank for monitoring the air temperature nearby. Along the east side of the upstream panel of the No.11 buttress, 14 thermometers (TS11085-TS11124) are installed to monitor the water temperature at different elevations. Two thermometers (T_wall1, T_wall2) are installed to monitor the temperature inside and outside the insulation layer on the west wall of No.21 buttress respectively, and so it is for the insulation layer on the east side of No.21 arch (T_arch1, T_arch2). In order to monitor the concrete and air temperature in the No.13 buttress, 36 thermometers are installed in the buttress concrete and on the inner surface of the buttress, which is shown in Fig. 3. The thermometers numbered T13-1–T13-16 are newly added in this reinforcement, and the thermometers numbered T13011–T13102 are original.

**Figure 4:**
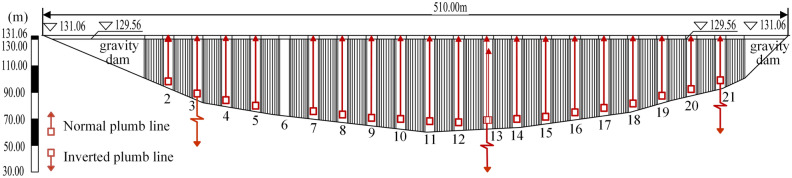
Layout of dam displacement monitoring points (unit: m).

As is shown in Fig. 4, 20 normal plumb lines (PL2–PL5, PL6–PL12, PL13-1, PL13-2, PL14–PL21) used to monitor dam crest displacement are installed in No. 2–5, 7–21 buttresses, among which there are two normal plumb lines in No. 13 buttress (cf. Fig. 3). Three inverted plumb lines (IP3, IP13, IP21) used to monitor foundation deformation are installed in No.3, 13, and 21 buttresses.

## The monitoring results

### Water level & temperature

**Figure 5:**
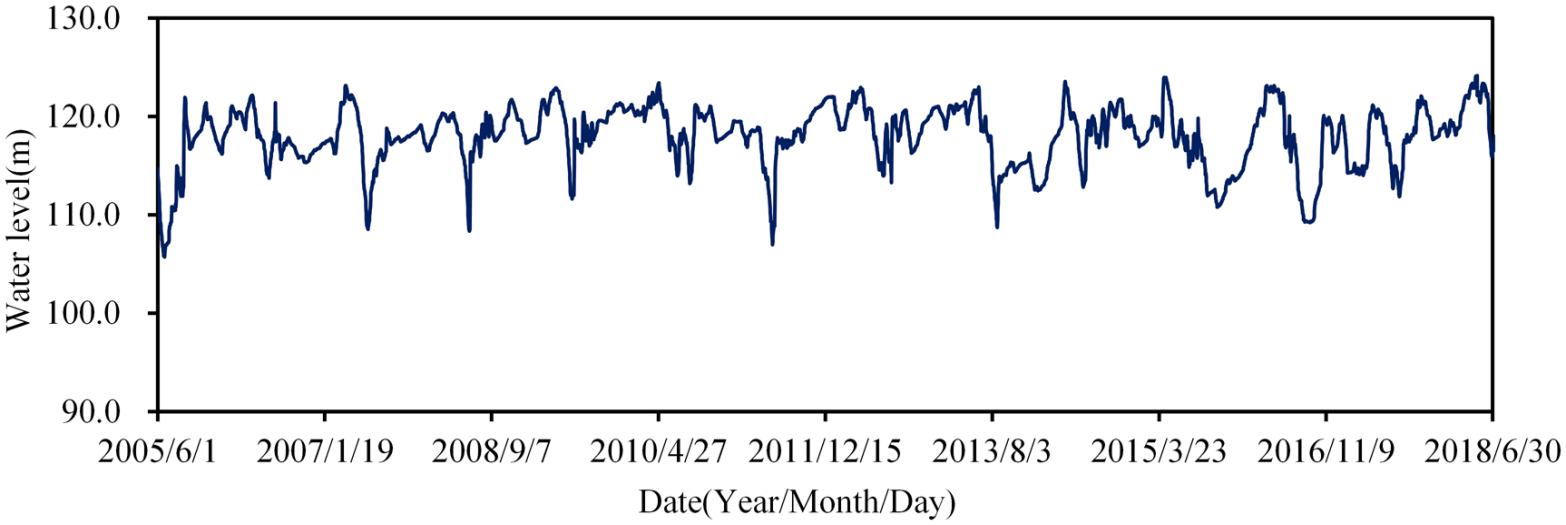
Water levels of Foziling reservoir.

Figure 5 shows the water level fluctuation after the reinforcement. For optimal irrigation and electricity generation, the water level is operated at elevations between 105.0 and 125.0 m.

**Figure 6:**
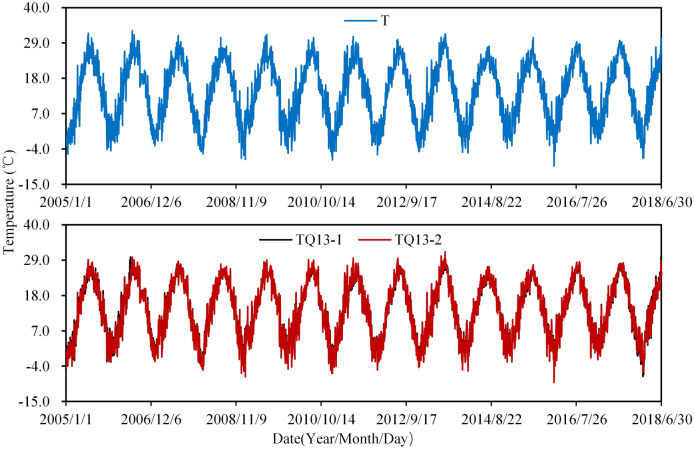
Measured daily air temperature.

**Figure 7:**
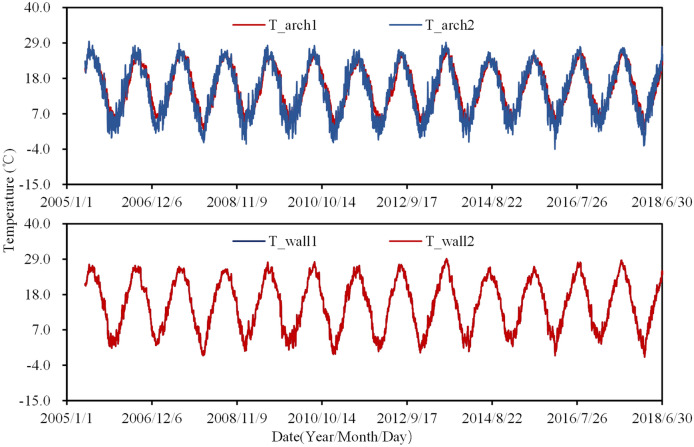
Measured temperature inside and outside the insulation layer.

The monitoring data of air temperature is shown in Fig. 6. It can be found that air temperatures are significantly correlated with the seasons. In winter, the temperature near the downstream of the arch is lower than that on the upstream side, but in summer it is the opposite. From the data of thermometers inside and outside the insulation layer shown in Fig. 7, it can be seen that the annual variation of the temperature inside the insulation layer is smaller than that outside, and the time response of the temperature in the insulation layer is a little hysteretic than that outside. This phenomenon illustrates that the insulation layer has good thermal insulation.

**Figure 8:**
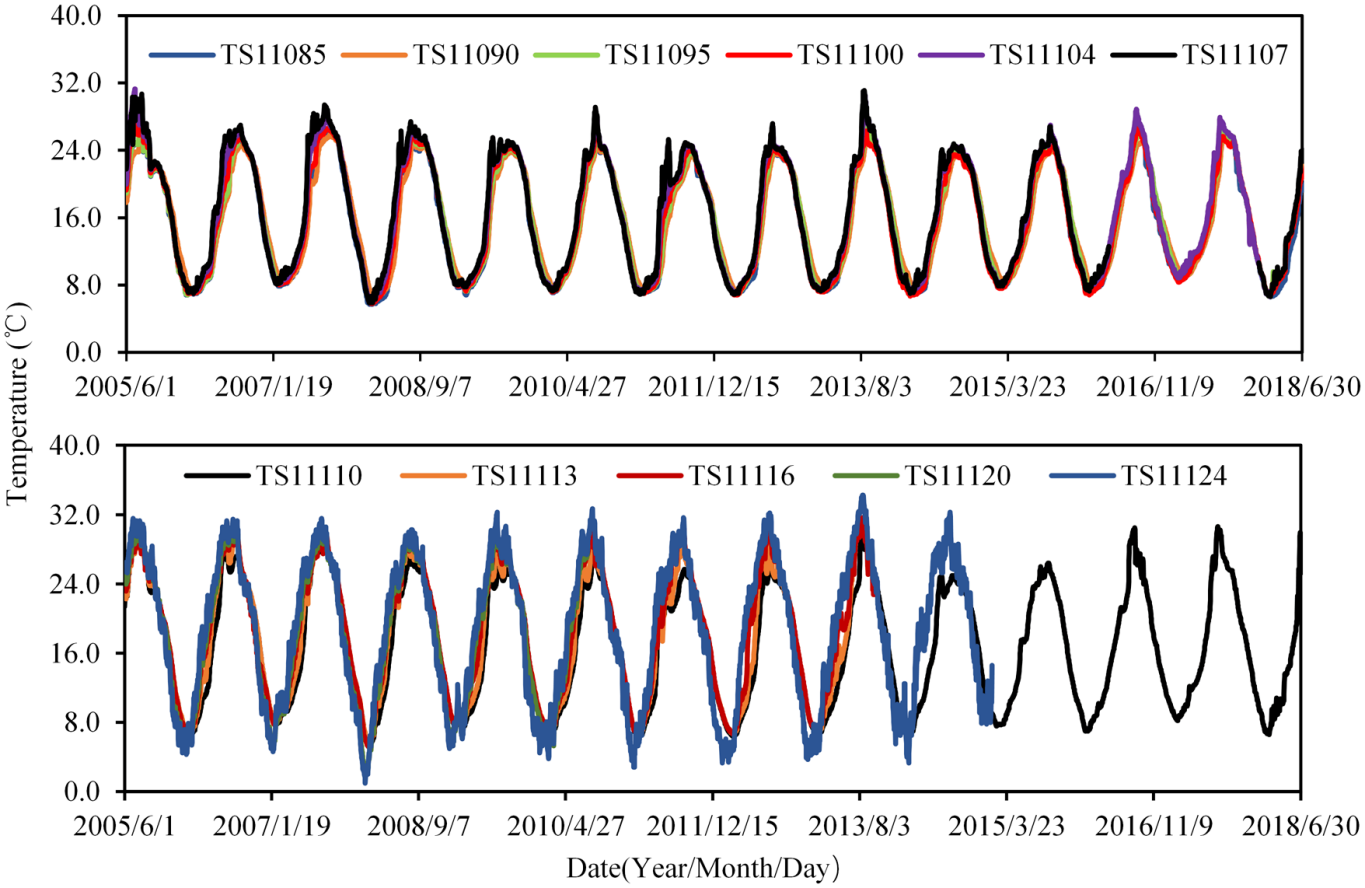
Water temperature at different elevations of Foziling reservoir.

**Figure 9:**
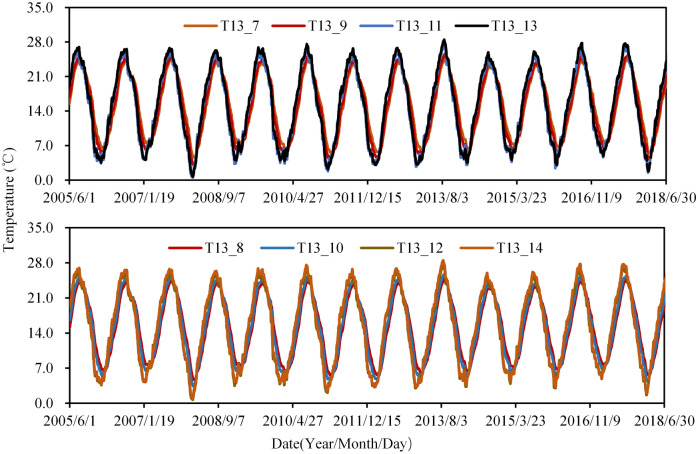
Air temperature and concrete temperature at selected typical measured points.

Figure 8 shows the water temperature at different elevations of the reservoir during the operation. It can be found that the greater the annual variation of temperature occurs at a higher elevation. The air temperature and concrete temperature of selected typical observation points are shown in Fig. 9. It can be seen that the annual variation of temperature near the buttress surface tends to be greater than those in the buttress, and the time response of the temperature upstream is a little more hysteretic than that close to the buttress surface. What is needed to point out is that some thermometers are not working, so data from 57 thermometers are selected as input temperature data for the establishment of HTT and MLSR models.

### Displacements

In this paper, dam displacements refer to the horizontal displacements of the buttress along with upstream and downstream (i.e., radial displacements) and the left–right displacements of the buttress along the left and right bank (i.e., tangential displacements). According to the buttresses' spatial orientation, the displacement observation points can be grouped into three parts: riverbed observation points (PL8–PL16), left bank observation points (PL2–PL5,) and right bank observation points (PL17–PL21). Since there are many displacement measuring points, typical observation points are selected for display and analysis.

**Figure 10:**
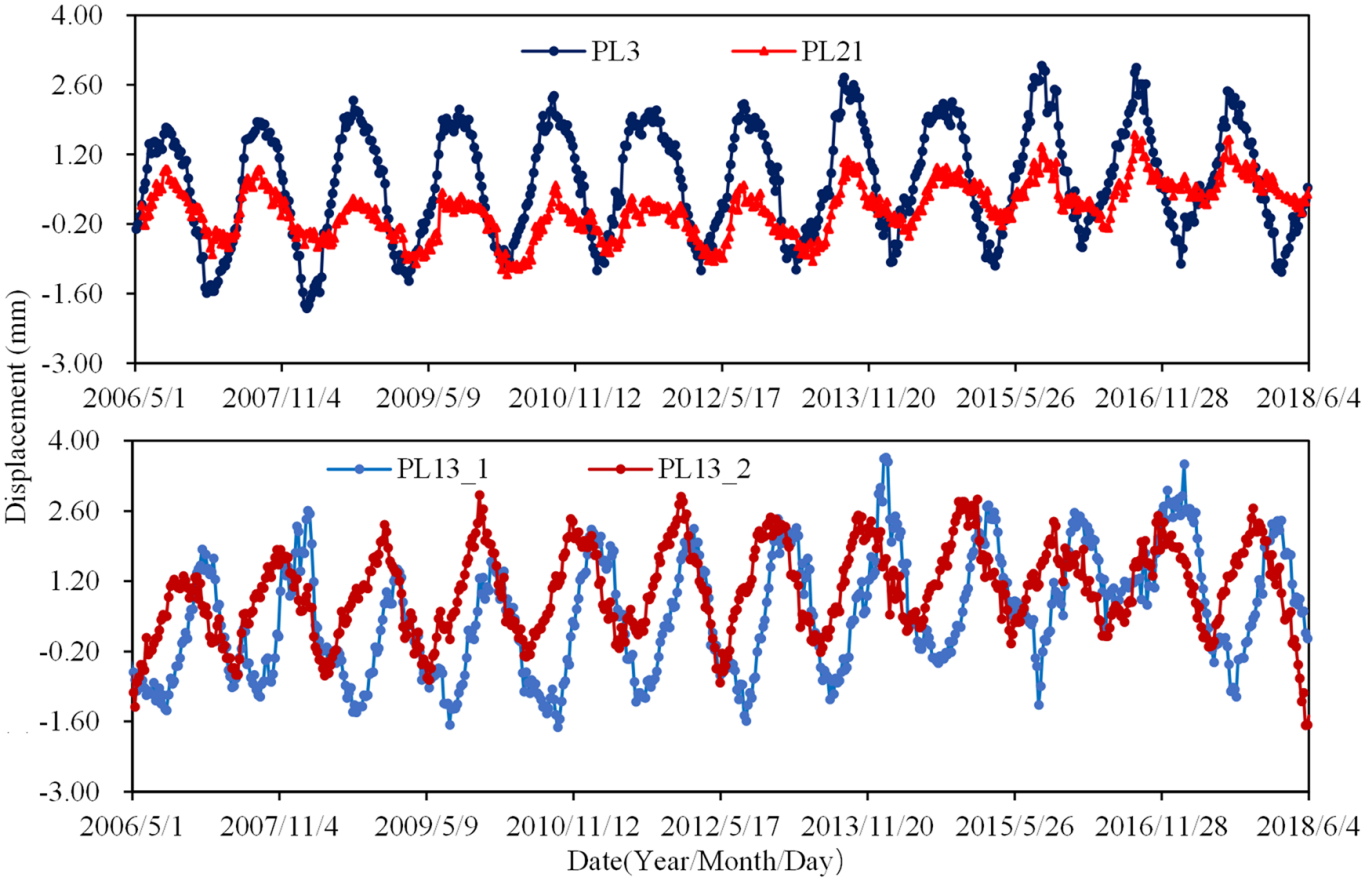
Radial displacements of the No. 3,13 and 21 buttresses.

Figure 10 shows the radial displacements of the No. 3, 13, and 21 buttresses over time. It can be seen that: (1) There is a significant correlation between the radial displacements and season. (2) The radial displacements of the buttresses on the riverbed (e.g., PL13_1 and PL13_2) are larger than those on the bank (e.g., PL3 and PL21). (3) The radial displacements of the buttress on the right bank (e.g., PL21) are smaller than those on the left bank (e.g., PL3). (4) The variances of the radial displacements at PL3, PL13_1, PL13_2, and PL21 are approximately 5.0 mm, 3.5 mm, 4.5 mm, and 2.5 mm, respectively.

**Figure 11:**
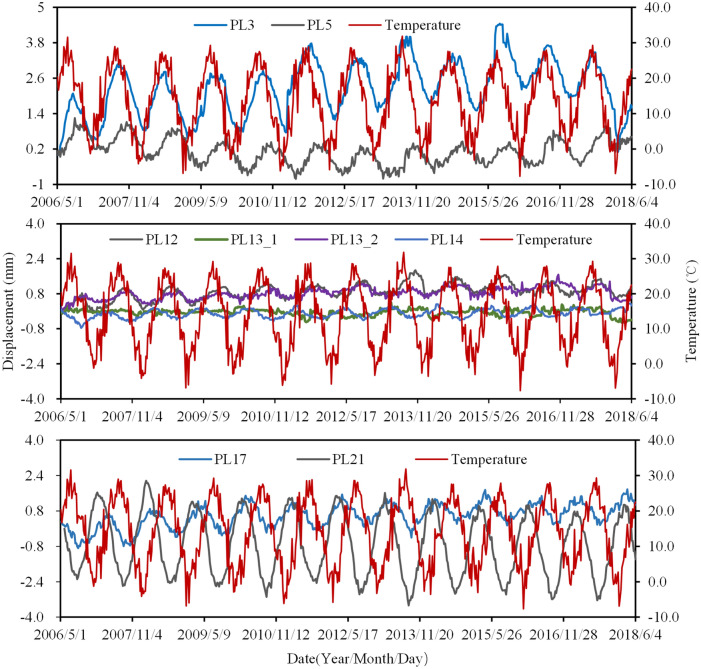
Tangential displacements of the No. 3,13 and 21 buttresses.

Figure 11 shows the tangential displacements of the buttresses over time. It can be seen as follows: (1) Taking the buttresses riverbed (No. 8–16 buttresses) as the midpoint, the tangential displacements of the buttresses on the left bank (No. 2–6 buttresses) are closely and positively correlated with temperature, while they are the opposite to the tangential displacements of the buttresses on the right bank (No. 17–21 buttresses). (2) The tangential displacements are cyclical, especially for the buttresses on banks, and the closer to the banks, the greater displacements occur. (3) The variances of the tangential displacements at PL3, PL5, PL12, PL13_1, PL13_2, PL14, PL17, and PL21 are approximately 5.0 mm, 2.0 mm, 1.0 mm, 1.5 mm, 1.0 mm, 1.0 mm, 2.0 mm, and 6.0 mm, respectively.

A large number of monitoring instruments provide a guarantee to master the change of water level, temperature, and the dam health state at all times. Displacement is the comprehensive effect of water pressure, temperature and time. The high similarity between displacement and temperature indicates that displacement is greatly affected by temperature. Then how individual factor affects the displacement and how much it contributes to the displacement are studied in the following section.

## Statistical models for dam behavior monitoring

### Hydrostatic, temperature, time–displacement monitoring model

A dam health monitoring model based on displacement is an effective method to analyze and obtain the working state of dams. Dam displacement is composed of two parts, one is reversible displacement caused by the fluctuations of reservoir water level and temperature. And the other part called irreversible displacement is caused by creep, alkali-aggregate reaction and other inelastic effects ^6,29^. Therefore, the dam displacement can be written as expressed below:

1 $$\delta = \delta_{H} + \delta_{T} + \delta_{\theta }$$

where *δ* is the measured displacement; *δ*_*H*_ is the water pressure component; *δ*_*T*_ is the temperature component and *δ*_*θ*_ is the aging component.

### Water pressure component δ_H_

Water pressure component *δ*_*H*_ represents the displacement of the dam body and foundation caused by reservoir hydrostatic pressure, and it is calculated as

2 $$\delta_{H} { = }\sum\limits_{i = 1}^{n} {\alpha_{i} H^{i} }$$

where *α*_*i*_ is the statistical coefficient; *n* is taken as 3 for the gravity dam and 4 for the arch dam; *H* is the upstream water depth.

### Temperature component δ_T_

Temperature component *δ*_*T*_ describes the thermal displacement caused by the temperature variations of the dam body and foundation. So it is the best choice to take the measured temperature of the dam body and foundation as calculation factors. In practical engineering, there are the following two situations regarding the layout of thermometers:

(1) When sufficient thermometers are embedded in the dam body and foundation, temperature component *δ*_*T*_ can also be calculated with measured temperature in HTT models

3 $$\delta_{T} = \sum\limits_{{i{ = }1}}^{m} {b_{i} T_{i} } \quad {\text{or}}\quad \delta_{T} = \sum\limits_{{i{ = }1}}^{{m_{1} }} {b_{1i} \overline{T}_{i} } + \sum\limits_{{i{ = }1}}^{{m_{1} }} {b_{2i} \beta_{i} }$$

where *b*_*i*_, *b*_*1i*_, *b*_*2i*_ are the statistical coefficients; m and m_1_ are the number of the effective thermometers and the layers equipped with thermometers, respectively; *T* is the measured temperature; $\overline{T}$ and *β* are the mean temperature and gradient of each layer, respectively.

(2) When there are no thermometers or few thermometers in the dam body and foundation, thermal displacement is generally assumed to follow the season evolution. So the hydrostatic-season-time (HST) model is widely used, in which the temperature component *δ*_*T*_ is usually simulated with a one-year or half-year period harmonic function

4 $$\delta_{T} = \sum\limits_{i = 1}^{{m_{2} }} {[b_{1i} \sin (is) + b_{2i} \cos (is)]}$$

where *m*_*2*_* is taken as 1*or2; *b*_*1i*_ and *b*_*2i*_ are the statistical coefficients; $s = 2\pi t/365$, and *t* is the number of accumulated days from the initial monitoring date.

The temperature field of the dam is essentially caused by the alternation of temperature with the seasons during the operation period, so the thermal displacement can be also calculated by the measured air temperature^1,4,21^. Due to the heat conduction effect, there is a temporal hysteresis in the response of concrete temperature to ambient air temperature change. When the measured air temperature is used to simulate the thermal displacement, the temperature component *δ*_*T*_ can be expressed as

5 $$\delta_{T} = \sum\limits_{{i{ = 0}}}^{d} {b_{d} T_{d} }$$

where *b* and *d* are the statistical coefficients; T is the measured or average measured air temperature; *d* is the number of temperature factors. Because of the thin shape of the multi-arch dam, the influence of air temperature a month ago is accounted for in this study. In order to simplify the calculation, *T*_*d*_ is set as *T*_*0*_, *T*_*1*_, *T*_*2*_, *T*_*3–5*_, *T*_*6–15,*_ and *T*_*16–30*_, where *T*_*0*_, *T*_*1*_, *T*_*2*_ are the measured air temperature of the monitoring day, the previous 1 and 2 days, respectively; *T*_*3–5*_, *T*_*6–15*_ and *T*_*16–30*_ are the average air temperature of the previous 3 to 5, 6 to 15 and 16 to 30 days, respectively. In this case, *d* is taken as 6.

### Aging component δ_θ_

Aging component *δ*_*θ*_ represents the time effects, it is usually expressed as

6 $$\delta_{\theta } { = }c_{1} \theta + c_{2} \ln \theta$$

where *θ* = *t/100*, *t* is the number of days since the beginning of the analysis, and c_1_ and c_2_ are coefficients.

## Multiple linear stepwise regression

It is necessary to point out that the harmonic function in HST models cannot effectively interpret the thermal displacement caused by the short-term dynamic temperature variations. Hence, based on Eqs. (1), (2), (3), (5), and (6), the HTT displacement health monitoring model of multi-arch can be expressed in the following two formulations:

7 $${\mathbf{M}}_{{\mathbf{1}}} : \delta { = }a_{0} { + }\sum\limits_{{i{ = }1}}^{4} {a_{i} (H_{i}^{i} - H_{0}^{i} )} { + }\sum\limits_{{\text{j = 1}}}^{m} {b_{j} (T_{j} - T_{0j} )} + c_{1} (\theta - \theta_{0} ) + c_{2} (\ln \theta - \ln \theta_{0} ){ + }\delta_{{0}}$$

8 $$\begin{gathered} {\mathbf{M}}_{{\mathbf{2}}} : \quad \delta { = }a_{0} { + }\sum\limits_{{i{ = }1}}^{4} {a_{i} (H_{i}^{i} - H_{0}^{i} )} { + }\sum\limits_{{k{ = 1}}}^{n} {w_{k} (T_{wk} - T_{w0} )} + b_{0} (T_{0} - T_{0}^{0} ){ + }b_{1} (T_{1} - T_{1}^{0} ) + b_{2} (T_{2} - T_{2}^{0} ){ + }b_{3} (T_{3 - 5} - T_{3 - 5}^{0} ) + b_{4} (T_{6 - 15} - T_{6 - 15}^{0} ) \\ + b_{5} (T_{16 - 30} - T_{16 - 30}^{0} ) + c_{1} (\theta - \theta_{0} ) + c_{2} (\ln \theta - \ln \theta_{0} ){ + }\delta_{{0}} \\ \end{gathered}$$

**M**_**1**_ and **M**_**2**_ are the HTT displacement health monitoring models based on measured air temperature and the temperature of the dam body and foundation respectively**.** Where $T_{w}$ is the water temperature at different elevations of the reservoir; *a*_0_ is a constant; *δ*_*0*_, *H*_*0*_ are the measured displacement and upstream water depth of the initial date in the modeling series, respectively; $T_{w0}$, $T_{0j}$, $T_{0}^{0}$, $T_{1}^{0}$, $T_{2}^{0}$, $T_{3 - 5}^{0}$, $T_{6 - 15}^{0}$, $T_{16 - 30}^{0}$, $\theta_{0}$ and $\ln \theta_{0}$ are the temperature and aging factors of the initial date in the modeling series, respectively.

If the random error is considered, Eqs. (7) and (8) extend to a generalized form of an MLSR model, which can be written in matrix notation as

9 $${\mathbf{D}} = {\mathbf{X\alpha }} + {{\varvec{\upvarepsilon}}}$$

where ${\mathbf{D}}$ is an (*m* × 1) dependent variables vector of the dam displacement and *m* is the number of displacement measuring points. ${\mathbf{X}}$ is an (*m* × (*i* + *j* + 3)) matrix of the independent variables expressed in $\delta_{H}$, $\delta_{T}$, and $\delta_{\theta }$. ${{\varvec{\upalpha}}}$ is a ((*n* + *i* + 3) × 1) vector of the regression coefficients. ${{\varvec{\upvarepsilon}}}$ is (*m* × 1) residual error between the measured displacements and the model predicted displacements.

The solving steps of the MLSR model are as follows:(1) The optimal selection of model factors is started with no variables in the model, testing the addition of each variable by *F-tests*. (2) The variables are added if their inclusion gives the most statistically significant improvement of the fit. (3) This process is repeated until none improves the model to a statistically significant extent. (4) The regression coefficients of Eq. (9) can be obtained by the least square method.

The above solutions can be realized by MATLAB programs. Moreover, the water pressure component $\delta_{H}$, the temperature component $\delta_{T}$_*,*_ and the aging component $\delta_{\theta }$ can also be obtained. Thus, the Foziling multi-arch dam behavior can be further analyzed.

The performance criteria for model performance evaluation include the coefficient of determination ($R$), the mean absolute error ($AE_{mean}$), and the root means squared error ($RMSE$).

10 $$R = \sqrt {\frac{{\sum\limits_{i = 1}^{n} {\left( {\hat{\delta }_{i} - \overline{\delta }} \right)} }}{{\sum\limits_{i = 1}^{n} {\left( {\delta_{i} - \overline{\delta }} \right)} }}}$$

11 $$AE_{mean} = \frac{1}{n}\sum\limits_{i = 1}^{n} {\left| {\delta_{i} - \hat{\delta }} \right|}$$

12 $$RMSE = \sqrt {\frac{1}{n}\sum\limits_{i = 1}^{n} {\left( {\delta_{i} - \hat{\delta }} \right)^{2} } }$$

where *n* is the number of samples; $\delta_{i}$ and $\hat{\delta }_{i}$ are the measured and calculated displacements, respectively; and $\overline{\delta }$ is the mean value of measured displacement.

## The behavior of multi-arch dam displacement analysis by MLSR

**Figure 12:**
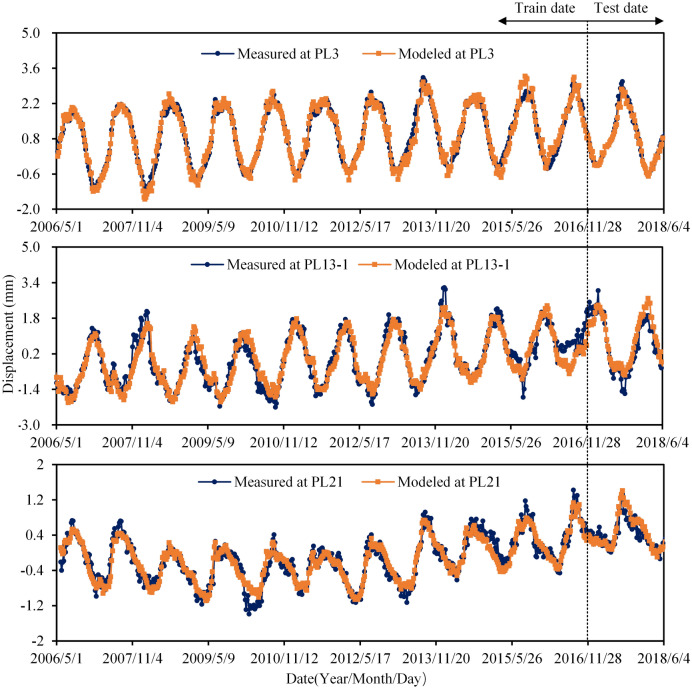
Monitoring date, MLSR model results for radial displacements at PL3, PL13-1 and PL21.

The data set of radial displacement, a period of 13 years (May 2006–July 2018, 624 observations) is selected for modeling. The observations from May 2006 to November 2016 are selected for training the model, while the observations from December 2016 to July 2018 are selected for testing the model. The data of radial displacement of observation points (PL3, PL13-1, PL21), taken as typical points, are selected for modeling and analysis. Considering thermometers measuring concrete temperature are only installed in buttress 13, based on Eq. (7), the HTT displacement health monitoring model (**M**_**1**_) based on measured concrete temperature is established. As for the No. 3, 21 buttresses, another HTT displacement health monitoring model (**M**_**2**_) is established based on measured air temperature and water temperature. According to the solving steps of the MLSR model, the optimal factors and the coefficients are listed in Table 1. And the performance parameters of the dam health monitoring models based on MLSR are listed in Table 2. The measured and modeled displacements are shown in Fig. 12. All of these indicate that the MLSR method performed well in fitting and predicting.

**Table 1:** Optimal factors and the coefficients of MLSR model.

PL3	Factors	H_4_	TS11085	TS11090	TS11110	T_0_	*lnθ*	*b*_0_			
	Coefficients	6.65e−8	0.13844	−0.11623	0.12052	0.00919	1.22760	−1.04294			
PL13-1	Factors	H_3_	H_4_	TS11110	T_arch1	T13091	T13082	T13102	*θ*	*lnθ*	*b*_0_
	Coefficients	1.79e−4	−2.91e−6	0.17626	−0.07677	0.15201	−0.4542	0.10773	0.15269	−4.35112	0.75102
PL21	Factors	H_2_	H_4_	TS11085	TS11100	TS11110	T_3–7_	T_8–15_	*θ*	*lnθ*	*b*_0_
	Coefficients	1.539e−3	-2.21e−6	0.08644	−0.11457	0.11481	−0.01527	−0.01891	0.08124	−2.25122	0.89741

**Table 2:** Performance parameters of MLSR model.

Measured points	Training			Test		
	*R*	*AE*_*mean*_	*RMSE*	*R*	*AE*_*mean*_	*RMSE*
PL3	0.9786	0.1704	0.2262	0.9316	0.1154	0.1713
PL13-1	0.9459	0.2907	0.3819	0.8852	0.4111	0.4943
PL21	0.9378	0.1410	0.1772	0.8859	0.1351	0.1816

**Figure 13:**
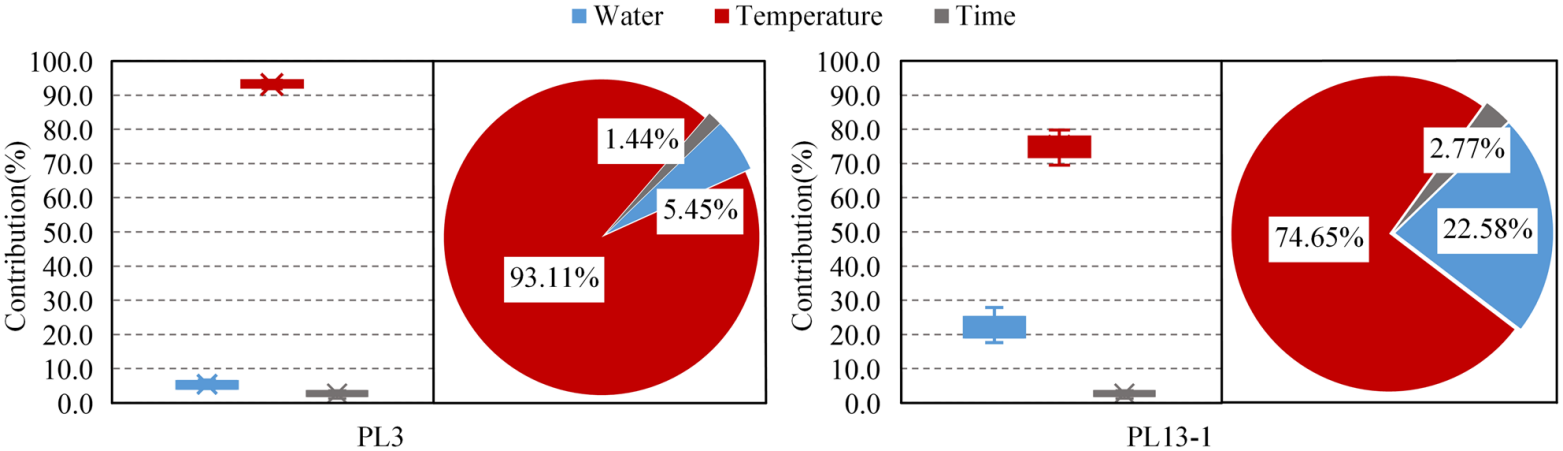
Contributions of water level, temperature and time to the radial displacement.

The water pressure component, temperature component and aging component of displacement observation points (PL3, PL13-1) are calculated based on Eqs. (2)–(6). Subsequently, contributions of water level, temperature and time to the annual amplitude of displacement are also obtained. Figure 13 shows the contributions of each component to the annual amplitude of the radial displacements from 2007 to 2017. It can be seen that: (1) In the annual amplitude of the radial displacements of the No. 3 buttress, the water pressure component accounted for 4.13–6.66%, the temperature component accounted for 91.89–94.37%, and the aging component accounted for 1.3–1.5%, with the average values of 93.11%, 5.45% and 1.44%, respectively. (2) In the annual amplitude of the radial displacements of No.13 buttress, the water pressure component accounted for 18.50–27.90%, the temperature component accounted for 69.58–79.82%, and the aging component accounted for 1.21–3.43%, with the average values of 22.58%, 74.65% and 2.77%, respectively. (3) The radial displacements of the buttresses on the riverbed are more affected by water pressure but less affected by temperature than buttresses on the banks, and there is little difference in the effect of time on them. (4) The radial displacement is the most affected by temperature, followed by water level and then time.

**Figure 14:**
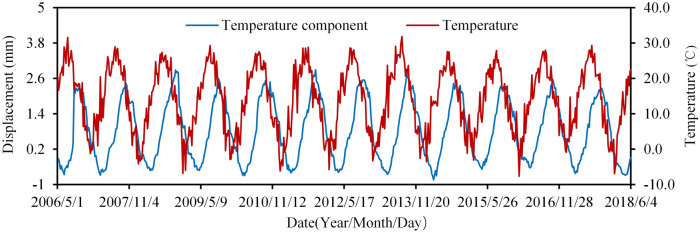
Measured temperature component and air temperature at PL13-1.

Figure 14 shows the relationship between air temperature and temperature component separated based on the HHT model at PL13-1. It illustrates how temperature affects radial displacement, that is, the crest is displaced downstream when the temperature rises, and the crest is displaced upstream when the temperature drops. The change of radial displacement lags the change of air temperature. Herein, the effect of temperature is the combination of temperature changes in the arches and buttresses. However, when they are viewed separately, their respective effects on the dam displacement are quite different.

**Figure 15:**
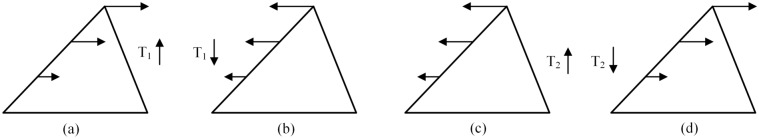
Influence of temperature variation on radial displacement: (**a,b)** are the temperature rise and fall of the arch, respectively; (**c,d)** are the temperature rise and fall of the buttress, respectively.

As for the arches, when the temperature rises, the buttresses are pushed downstream by the thrust induced by the thermal expansion of the connected arches (cf, Fig. 15a), while the temperature drops, the buttresses are pulled upstream due to the shrinkage of arches (cf, Fig. 15b). As for the buttresses, the crest of the buttress is displaced upstream when temperature rises (cf, Fig. 15c), and the crest of the buttress is displaced downstream when temperature drops (cf, Fig. 15d), which is the same to arch dams and gravity dams.

**Figure 16:**
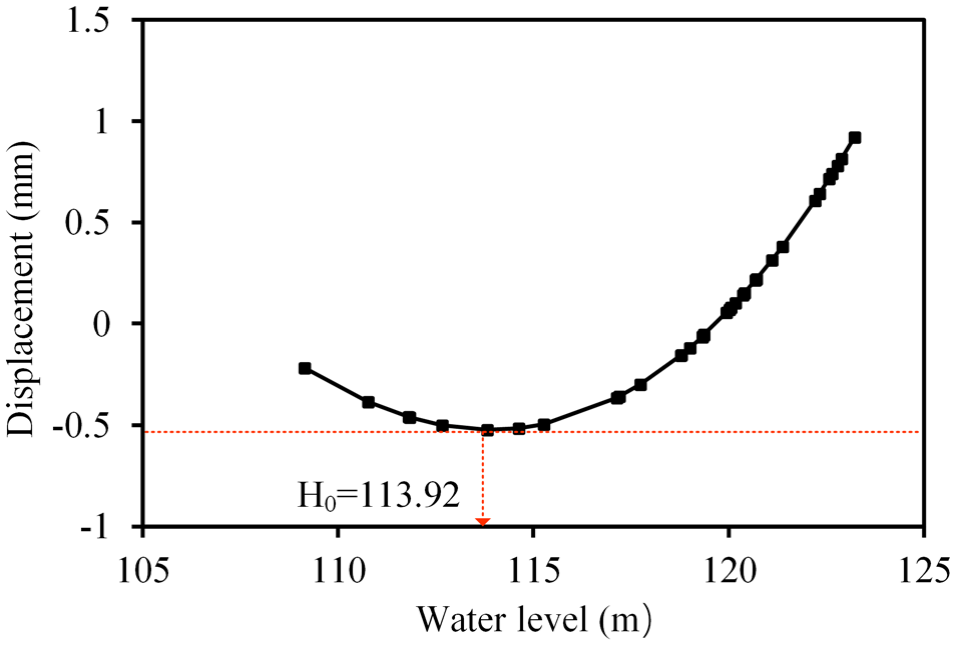
The relationship between water level and water pressure component at PL13-1.

As the proportion of the water pressure component is relatively small, the water pressure component separated from the model established by using the whole data is biased. Therefore, in order to highlight the effect of water pressure on the radial displacement as much as possible, the measured values corresponding to the air temperature between 20 and 23 °C are selected, and the water pressure component is separated by the MLSR method. Figure 16 reflects the relationship between the water pressure component and water level change. It can be seen that when the reservoir level is above *H*_*0*_ (113.92 m), the radial displacement is positively correlated with the water level, i.e., the crest is displaced downstream as the water level rises, and vice versa. While the water level is below *H*_*0*_, the radial displacement is negatively correlated with the water level, i.e., the crest is displaced upstream as the water level decreases.

**Figure 17:**
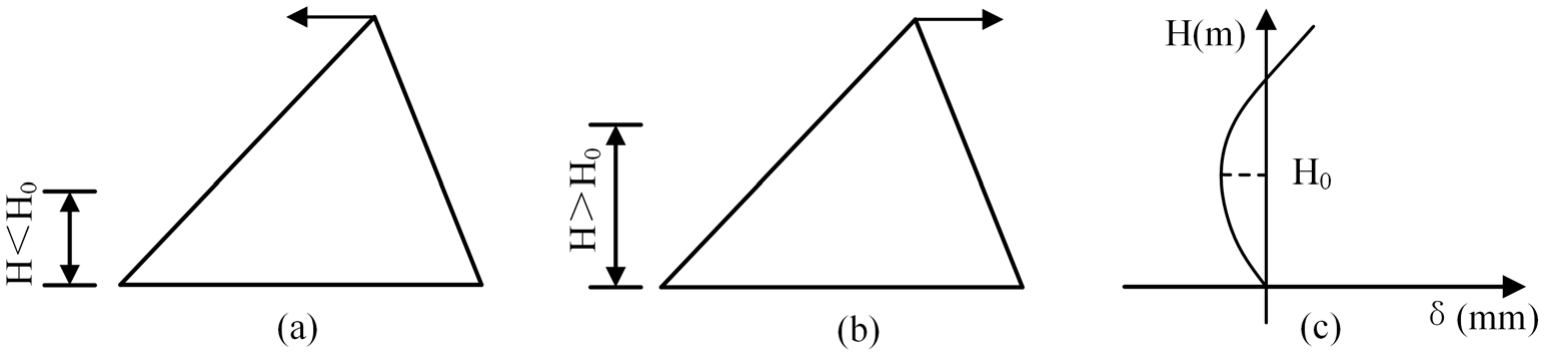
Influence of water level variation on radial displacement.

The reason is that when the water level is lower (*H* < *H*_*0*_) (cf, Fig. 17a), the resultant line of the action, which is transmitted from the upstream water pressure through the arch to the buttress, passes upstream of the midpoint of the buttress-foundation surface, a counterclockwise bending moment is generated, and the crest is displaced downstream. When the water level is higher (*H* > *H*_*0*_) (cf, Fig. 17b), the resultant action line passes downstream of the midpoint of the buttress-foundation surface, a clockwise bending moment is generated, and the crest is displaced downstream.

**Figure 18:**
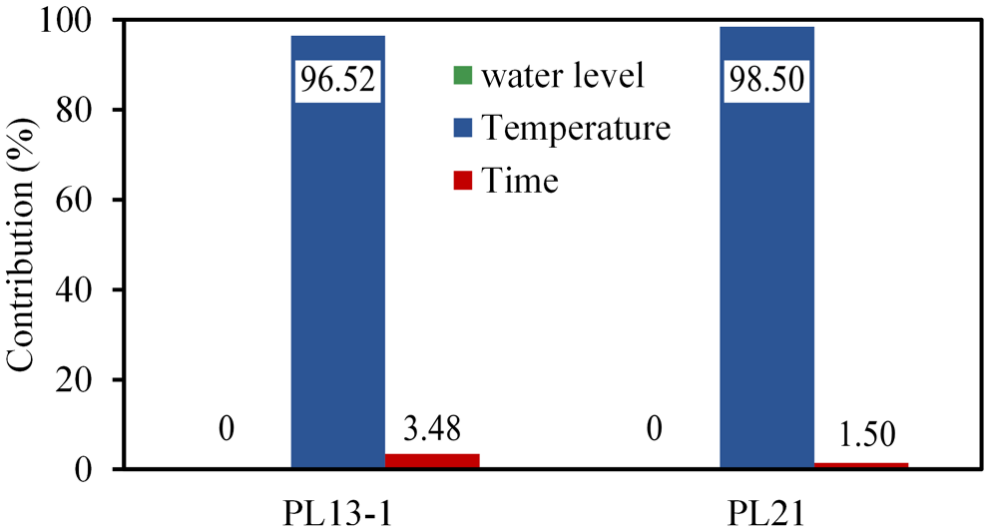
The average contributions of water level, temperature and time to the tangential displacement.

Similar to radial displacements analysis, the contributions of water level, temperature and time to the annual amplitude of tangential displacements (PL13-1, PL21) are calculated based on the HTT model. The average contributions of each component in the last 11 years (2007 to 2017) are shown in Fig. 18. It can be seen that: (1) In the tangential amplitude of the tangential displacements of No.13 and 21 buttresses, the temperature component accounted for 96.52% and 98.50% on average, respectively, and the aging component accounted for 3.48% and 1.50% on average, respectively. (2)The tangential displacement is almost entirely induced by temperature.

The direction of water pressure is perpendicular to that of tangential displacement, and the dam has been in operation for many years, so the water pressure and time have little effect on the tangential displacement. The effect of thermal expansion and contraction is the fundamental cause of dam tangential displacement. When the temperature drops, the arch shrinks, pulling the buttresses on both banks to the middle, so the buttresses on the left bank move towards the right bank, while the buttresses on the right bank move towards the left bank. When the temperature rises, the dam body is heated and expanded, pushing the buttresses to the banks, so the buttresses on the left bank move towards the left bank, while the buttresses on the right bank move towards the right bank.

The above analysis results illustrate that temperature has the greatest influence on displacement. Therefore, the observation should be increased during the period when the temperature is prone to sudden increase or decrease. Due to the superimposition effect of tangential displacement, more attention should be paid to the arches on the right and left banks in case of cracking generation. Foziling multi-arch dam is a kind of light and thin structure, the drastic temperature change will inevitably lead to great temperature stress. Then, simulating the stress state of the dam under variable temperature would be of great significance to further evaluate the dam safety.

## Numerical analysis of multi-arch dam stress behavior

### Numerical model

**Figure 19:**
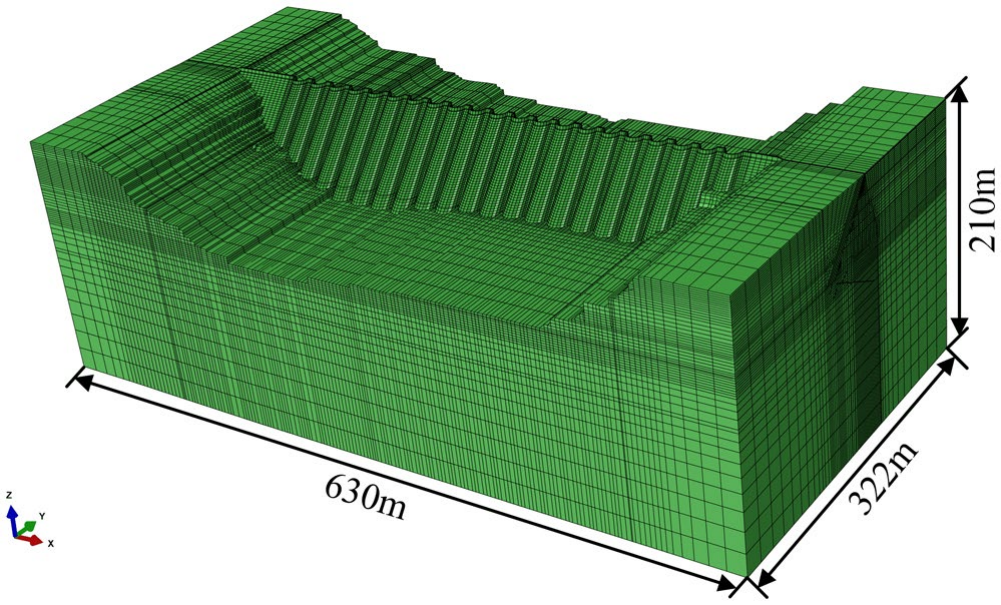
Numerical model of Foziling dam.

The finite element method (FEM) numerical model is an effective technique to study dam behavior and investigate dam safety. As is shown in Fig. 19, the three-dimensional finite element mesh of the Foziling multi-arch dam is established, in which 508,094 elements and 568,417 nodes are included. In the numerical simulation, an elastic model is used for the dam-foundation system, and the stress safety state of the elements is assessed compared with allowable tensile strength and compressive strength obtained based on the relative code.

The MLSR model has shown that, compared with the water pressure and temperature component, the aging component is negligibly small and tends to be stable. Hence, only the water level and temperature variations are considered as the main loads after the geostatic state of the model.

Parameters about the dam body concrete and foundation rock, determining the stress strength and deformation characteristic, are important foundations for obtaining reliable results by numerical modeling. The appropriate mechanical and thermodynamic parameters^35^ in the FEM simulation are summarized in Table 3.

**Table 3:** Mechanical and thermodynamic parameters for numerical simulation.

Materials		Poisson ratio	Elastic modulus $({\text{GPa}})$	Density $({\text{kg/m}}^{{3}} )$	Thermal conductivity kJ/(m·d·°C)	Specific heat kJ/(kg·°C)	Linear expansion coefficient (10^–6^)
**Concrete**							
Reinforced concrete partition		0.167	32.5	2551	815.616	0.95	8.0
Dam concrete		0.167	32.8	2469	929.664	0.97	8.0
Steel fiber reinforced concrete		0.167	30.8	2318	875.232	0.97	8.0
Backfill concrete		0.167	22.2	2400	929.664	0.97	8.0
**Rock of foundation**							
AII_1_		0.2	20.0	2760	–	–	–
AII_2_		0.2	17.5	2750	–	–	–
AIII		0.2	10.0	2741	–	–	–
AIV		0.2	7.0	2733	–	–	–
**Thermal insulation**							
Foam polyurethane		–	–	–	3.672	2.0	–

Based on the characteristics and properties, the rock of foundation is divided into four categories: AII1, AII2, AIII and AIV.

The normal water level, design flood level, and check flood level of the dam are 125.56 m (in the future), 125.97 m, and 129.97 m, respectively. The multi-arch dam body is thin and the temperature field mainly depends on the periodic change of ambient air temperature, so there is no stable temperature field in the dam body. Therefore, the difference between the maximum monthly average temperature and the annual average temperature in the operation period is taken as temperature rise load. The difference between the minimum monthly average temperature and the annual average temperature is taken as temperature drop load. The numerical simulation procedure can be divided into three parts: (1) A heat transfer model is established, in which the parameter equivalence method^36^ is used to simulate the thermal insulation layer. (2) Based on the measured data including water and air temperature, the highest temperature field, the lowest temperature field, and the quasi-stable temperature field are obtained by applying different boundary temperatures. (3) The linear expansion model is established with the same mesh as the heat transfer model. After the model is reached equilibrium under gravity, water pressure is applied at the upstream surface by hydrostatic loading. The temperature fields are set as predefined fields in each calculation step. Then the safety of the dam is evaluated based on the stress results of the numerical simulation.

## Analysis of the numerical results

High water levels, high water temperature, and sudden temperature rise and fall are the disadvantageous loading cases for the dam safety because of the temperature susceptibility of the dam and the small storage capacity. In the simulation, the normal storage level at 125.56 m, the checked flood level at 129.96 m, and high and low temperatures are considered. Here, high and low temperatures refer to the average monthly maximum and minimum temperature. Thus, there are four loading combinations, which are listed in Table 4.

**Table 4:** Loading combinations for numerical simulation.

Loading combinations	Water loading (m)	Season	Temperature loading
C1	Normal water level (125.56 m)	Winter	Temperature drop
C2		Summer	Temperature rise
C3	Check flood level (129.96 m)	Winter	Temperature drop
C4		Summer	Temperature rise

**Figure 20:**
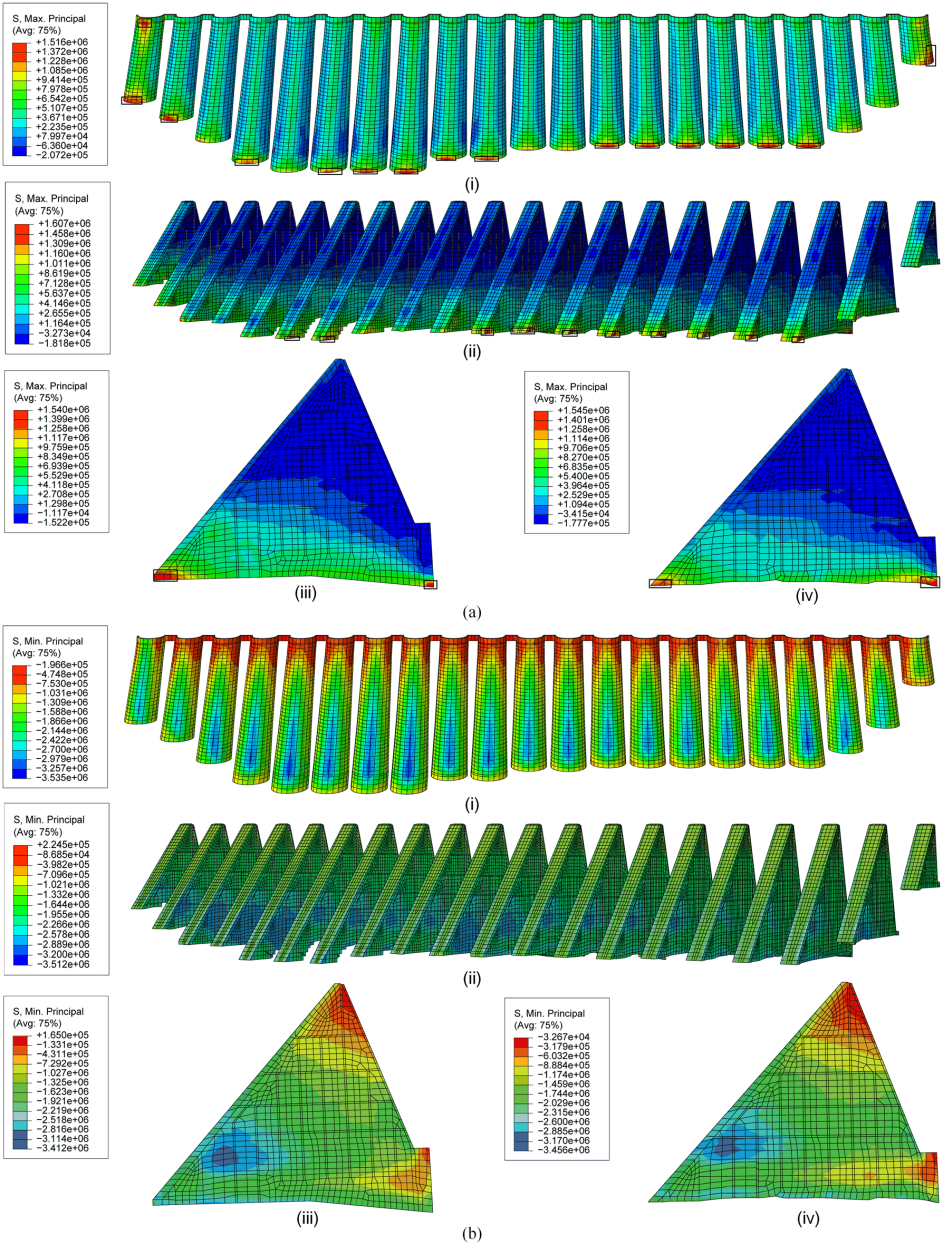
Principal stresses under loading C1: (**a**) maximum and (**b**) minimum principal stresses on (i) arches, (ii) buttresses, (iii) No. 12 buttress and (iv) No. 13 buttress (unit: Pa).

**Figure 21:**
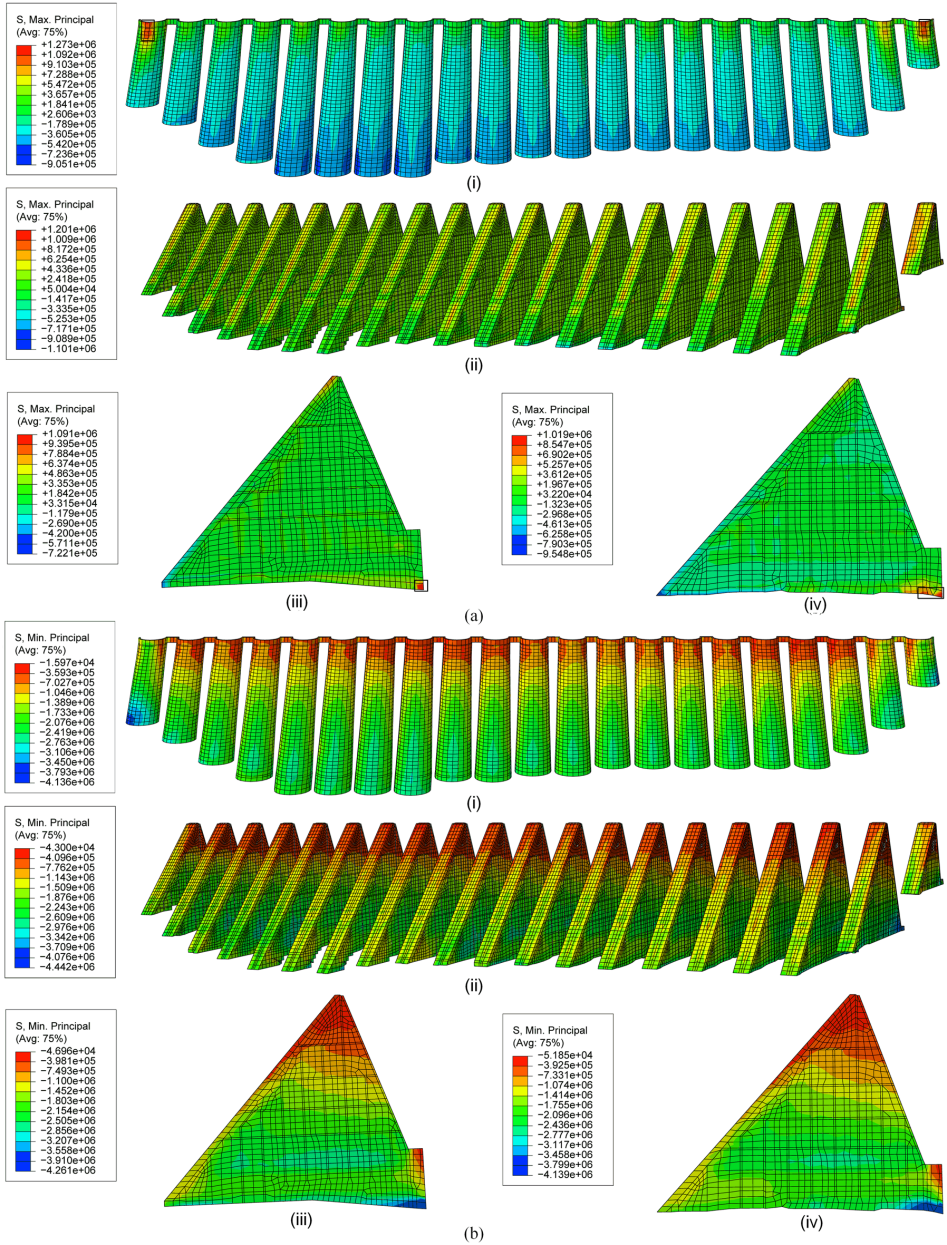
Principal stresses under loading C2: (**a**) maximum and (**b**) minimum principal stresses on (i) arches, (ii) buttresses, (iii) No.12 buttress and (iv) No.13 buttress (unit: Pa).

It is assumed that compressive stress is negative and tensile stress is positive. The principal stresses distributions in loading combinations (C1 and C2) are shown in Figs. 20 and 21 respectively, in which No.12 and 13 buttresses are taken as examples. It can be seen as followings:

1. The tensile stress in most areas of the arches and buttresses is less than the allowable tensile stress (0.9 Mpa) of the dam concrete in C1, except for No. 2 and 22 arches. In the middle and lower areas of No. 12 and 13 buttresses, tensile stress occurs. Due to the constraint of the foundation, the maximum tensile stresses of No. 12 and 13 buttresses reach 1.54 Mpa and 1.55 Mpa, respectively (cf. Fig. 20a), exceeding the allowable tensile stress, but less than the ultimate tensile stress (3.53 Mpa). It also occurs in the bottom region of the arch.
2. The maximum compressive stresses of No. 3–21 arches mainly occur at the elevation near 1/3 of the water depth in C1, and the maximum value reaches 3.54 Mpa, which is very similar to the distribution of the maximum compressive stress of buttresses. The maximum compressive stress of No. 12 and 13 buttresses are 3.41 Mpa and 3.46 Mpa, respectively (cf. Fig. 20b), which are much lower than the allowable compressive stress (12.45 Mpa).
3. The tensile stress of arches and buttresses generally decreases in C2 compared with that in C1. The greater tensile stress occurs in the middle of the buttress upstream panels, and none of them exceeds the allowable tensile stress. Moreover, the stress concentration in the contact part between arches and foundation disappears, but it still exists in the buttress toe (cf. Fig. 21a). The maximum compressive stress of No. 12 and 13 buttresses are 4.26 Mpa and 4.14 Mpa, respectively.
4. Greater tensile stress occurs in the middle of the crest of No. 2 and 22 arches in C1 and C2. And the maximum values reach 1.46 Mpa and 1.27 Mpa, respectively. Near the bottom of the side of No. 2 and 22 arches connected to the gravity dam, greater tensile stress (1.52 Mpa) occurs in C1, while greater compressive stress (4.14 Mpa) occurs in C2.

The special load cases are also applied to the dam in C3 and C4. The principal stresses distributions are very similar to C1 and C2, but the stress values generally increase. It is not repeated here. The maximum tensile stresses and maximum compressive stresses are listed in Table 5.

**Table 5:** Maximum and minimum principal stresses in C3 and C4.

Loading combinations	Part	Maximum principal stress (Mpa)	Minimum principal stress (Mpa)
C3	No. 2 and 22 arches	1.25	3.15
	No. 3–21 arches	1.68	3.85
	All buttresses	1.84	3.76
	No. 12 buttress	1.79	3.61
	No. 13 buttress	1.77	3.59
C4	No. 2 and 22 arches	1.33	4.59
	No. 3–21 arches	0.92	3.86
	All buttresses	1.26	4.98
	No.12 buttress	1.10	4.75
	No.13 buttress	1.13	4.71

Due to the great tensile stresses that occurred close to the crown of No. 2 and 22 arches, as well as the foundation of the arches and buttresses in C1 and C3, exceeding the allowable tensile strength, the load case of *high water level* + *temperature drop* is regarded as the most disadvantageous operating load combination for the dam safety.

Compared with the simulated stress before reinforcement^35^, the stress is generally reduced, indicating that the safety of the dam is improved. The areas exceeding the allowable tensile stress are considered as the areas prone to damage, which are pointed out in the rectangular zones (cf. Figs. 20 and 21). Since the sensitivity of the multi-arch dam to temperature and the cumulative effect of deformation, the tangential displacements of No. 2 and No. 22 buttresses are greater than others. In addition, one end of the No. 2 and 22 arches is connected with the gravity dam, and the other end is connected with the buttress. The difference in stiffness between them is so great that the end connected to the gravity dam is more constrained and subjected to greater forces. Thus, as the water level and temperature change continuously, fatigue cracks tend to occur on the No. 2 and 22 arches. As a result, No. 2 and 22 arches and the bottom of arches and buttresses are considered as the weak parts of the dam, and more attention should be paid in the safety patrol.

Therefore, the areas of the No. 2 and 22 arches from the foundation to the 127.46 m elevation are reinforced by steel fiber shotcrete in the reinforcement. The areas, the upstream and downstream surface of the arches and its left and right extensions 0.5 m wide, were thickened by 0.15 m and 0.1 m, respectively. For the great tensile stress areas on the upstream concrete face and sidewalls of each buttress, steel fiber concrete was sprayed for thickening from the inside of the buttress. The upstream concrete face was thickened by 0.5 m, the bottom (below elevation 79.56 m) was thickened by 0.7 m, and the left and right side walls were thickened by 0.4 m. Moreover, the compressive strength of steel fiber concrete is 48.0Mpa, the tensile strength is 3.5Mpa and the bond strength with old concrete is 2.5–3.0Mpa^35^. These reinforcement projects are intended to prevent cracks.

## Conclusions

In this study, the deformation of Foziling multi-arch dam is characterized based on the monitoring data. The contributions of water level, temperature, and time to the radial displacement of the dam are quantified by the HTT model and MLSR method. Then the influence characteristics of water pressure and temperature on displacement are analyzed. Finally, a numerical model is established to investigate the stress state of the dam in different load cases. The main conclusions can be obtained from this study as follows:

1. Foziling multi-arch dam is in an elastic state after the last reinforcement based on the good performance of MLSR at typical displacement monitoring points. The influence characteristics of water level and temperature on dam displacement are interpreted by separating variables based on the HTT model. The buttress is displaced downstream (upstream) when the temperature rises (drops). As the water level rises from low to high, the relationship between the displacement and the water level is firstly negative and then positive.
2. Temperature contributes the most to the radial displacements of the buttresses, followed by water level, then time. The tangential displacement is almost entirely induced by temperature. It can be concluded that the multi-arch dam is quite sensitive to temperature fluctuation, and the drastic fluctuation of temperature is a disadvantageous factor for dam safety.
3. Greater tensile stresses mainly occur close to the crown of No.2 and 22 arches and the bottom of arches and buttresses, especially in low temperature and high water level load cases. In addition, fatigue damage may occur in areas with large stress variation, probably leading to crack initiation. Although the application of steel fiber reinforced concrete in the region of high stress can effectively alleviate the generation of fatigue cracks, more attention should be paid to the weak parts of the dam.
4. The results of displacements and stresses analysis illustrate that the dam is still in good operation since the last reinforcement, and the effectiveness of the reinforcement projects, lays a good foundation for future operation in higher water levels. On the other hand, it also shows that the methods proposed in this paper have the value of engineering popularization and application to other concrete dams.
